# The change of gut microbiota in MDD patients under SSRIs treatment

**DOI:** 10.1038/s41598-021-94481-1

**Published:** 2021-07-21

**Authors:** Yang Shen, Xiao Yang, Gaofei Li, Jiayu Gao, Ying Liang

**Affiliations:** 1 National Clinical Research Center for Mental Disorders, Peking University Sixth Hospital, Institute of Mental Health, Key Laboratory of Mental Health, Ministry of Health, Peking University, Haidian District, Huayuanbeilu 51, Beijing, 100191 China; 2 School of Clinical Medicine, Henan University of Science and Technology, Luoyang, China; 3 Department of Psychiatry, Beijing Hospital of Chinese Traditional and Western Medicine, Beijing, China; 4 School of Chemical Engineering and Pharmaceutics, Henan University of Science and Technology, No. 263, Kaiyuan Boulevard, Luolong District, Luoyang, 471023 Henan China

**Keywords:** Depression, Metagenomics

## Abstract

The alterations in the gut microbiota have been reported to be correlated with the development of depression. The purpose of this study was to investigate the changes of intestinal microbiota in depressed patients after antidepressant treatment. We recruited 30 MDD patients (MDD group) and 30 healthy controls (control group). The MDD group received individualized treatment with escitalopram at a maximum dose of 20 mg/day. After depressive symptoms improved to a HAMD scale score > 50%, a fecal sample was collected again and used as the follow-up group. The differences of gut microbiota between patients and controls, the characteristics of gut microbiota under treatment and the potential differences in metabolic functions were thus investigated. The Firmicutes/Bacteroidetes ratio was significantly different within three groups, and the ratio of follow-up group was significantly lower than those of the other two groups. Alpha diversity was significantly higher in MDD group than those of the other groups, and the alpha diversity was not significantly different between control and follow-up groups. The beta diversity of some patients resembled participants in the control group. The metabolic function of gut microbiota after treatment was still different from that of the control group. This study suggests that the intestinal flora of depressed patients has a tendency to return to normal under escitalopram treatment.

## Introduction

As a common mental disorder accompanied by high disability and suicide, major depressive disorder (MDD) has become a worldwide issue^1^. In recent years, several studies have shown the correlation between gut microbiota and the development of depression^2–4^. In several studies, MDD patients showed specific features of gut microbes differing from normal controls^5–8^. It has been reported that transplants of MDD patients' feces into mice could cause the depression-like behaviors, which used to establish animal models of MDD^5, 9, 10^. Moreover, it also observed that the fecal bacteria transplantation of patients could lead to the increase of microglial cell density and expression of IL-1 in the ventral hippocampus^11^. Taken together, the change of gut microbiota could be correlated with the occurrence of MDD.

At present, selective serotonin reuptake inhibitors (SSRIs) are widely used in clinical practice and have therapeutic effects in the treatment of depression^12^. Besides, several SSRIs drugs, including sertraline, fluoxetine, paroxetine and escitalopram, could present antibacterial effects directly^13, 14^. For example, staphylococcus and enterococcus are especially vulnerable to sertraline, fluoxetine and paroxetine^14–16^. Therefore, SSRIs have demonstrated both of antidepressant and antimicrobial properties^17^. Ramsteijn et al.’s study reported that fluoxetine treatment altered important features of this transition from pregnancy to lactation and led to the decreased fecal amino acid concentrations. Amino acid concentrations negatively correlated with the relative abundance of bacterial taxa such as Prevotella and Bacteroides^18^. McVey Neufeld et al*.* reported that intestinal exposure to SSRIs could increase the excitability of intrinsic primary afferent neurons in the intermuscle plexus and alter the alpha diversity of the intestinal microbiota^19^.

Overall, SSRIs could directly or indirectly influence the changes of gut microbiota which might play the key role in the development of MDD. The purpose of this study was to explore the difference of gut microbiota with first episode MDD and elucidate the changes of gut microbiota after treatment by SSRIs.

## Results

### Clinical data

In this study, 30 patients with drug-naive first-episode MDD and 30 healthy controls were recruited, respectively. There were no statistically significant differences between patients’ group and controls group in terms of age, gender, height, weight, and tobacco and alcohol consumption (*p* > 0.05) (Table 1). The MDD group received an individualized treatment and the maximum dose was 20 mg/day. The average dose of escitalopram was 16.33 ± 3.46 mg/day. Under escitalopram treatment, the mean time of the HAMD score decreased over 50% was 34.53 ± 5.18 days. All patients have response with 50% HAMD reduction.

**Table1:** Demographic characteristics of MDD and controls.

	MDD (n = 30)	Controls (n = 30)	*p* value
	M ± SD	M ± SD	
Age (years)	44.83 ± 11.00	43.97 ± 10.57	0.757
Gender (M/F)	13/17	15/15	0.605
Height (m)	1.68 ± 0.07	1.70 ± 0.05	0.171
Weight (kg)	67.83 ± 6.86	69.21 ± 7.14	0.447
BMI (kg/m^2^)	23.99 ± 2.05	23.83 ± 2.08	0.761
Tobacco (%)^a^	46.67%	30.00%	0.288
Alcohol (%)^a^	53.33%	33.33%	0.192

^a^Chi-square test; compared with HCs, *p* < 0.05; *BMI* body mass index.

### Sequencing data and bacterial taxonomic composition

#### Sequencing data

Total 4,790,651 original sequences were obtained from 90 samples. After double-end Reads splicing and filtering, a total of 4,444,748 Clean tags were generated. Each sample generated at least 12,039 Clean tags. Taxonomic annotation of OTUs was based on Silva (version 138.1) and UNITE (version 7.0) taxonomic databases.

#### Bacterial composition comparisons within three groups

**Figure 1:**
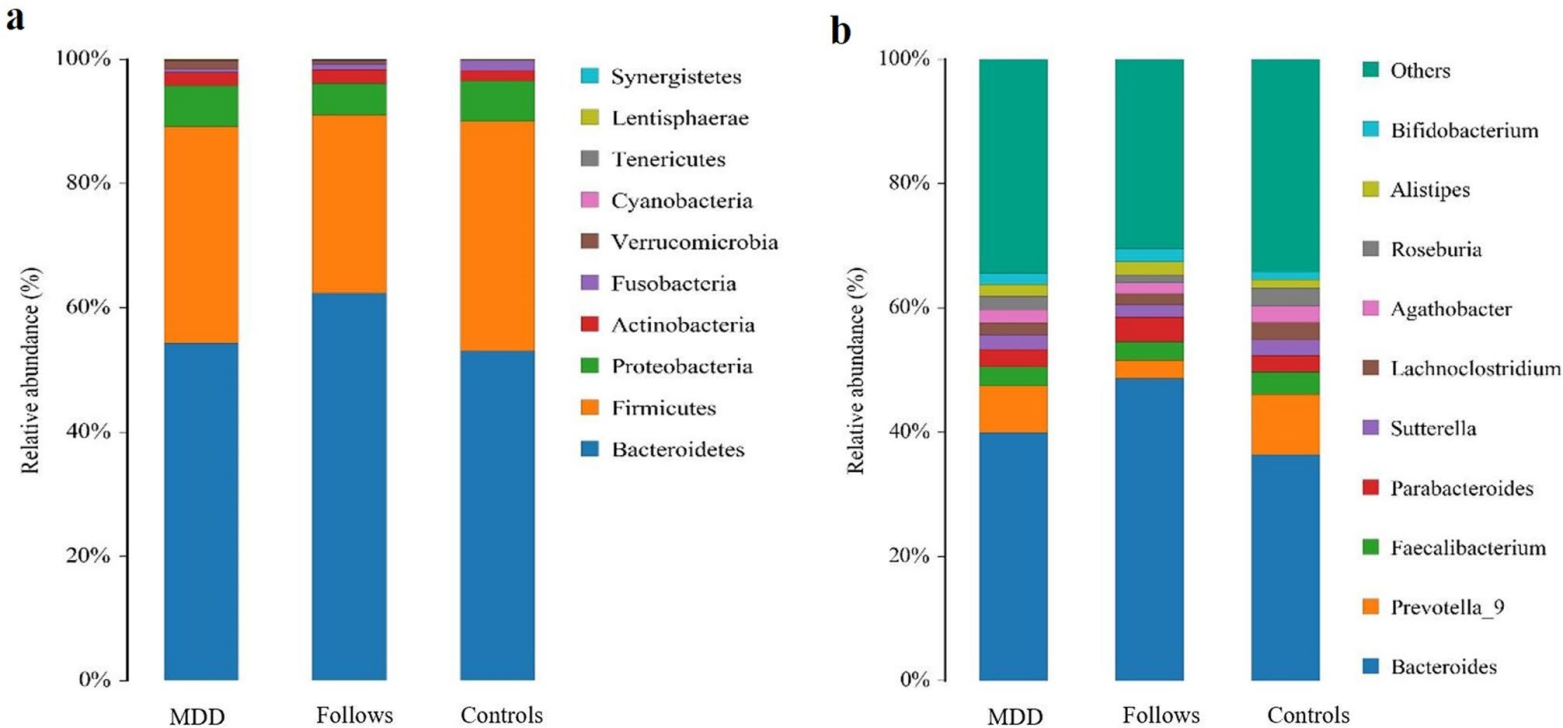
Histogram of species distribution. QIIME software was used to generate species abundance tables at different taxonomic levels, and R language tool was used to draw community structure charts at different taxonomic levels. (**a**) Relative proportions of species distribution at the phylum level; (**b**) relative proportions of species distribution at the genus level.

At phylum level, the dominated gut microbiota was composed by Bacteroidetes, Firmicutes, Proteobacteria and Actinobacteria in three groups. Bacteroidetes and Firmicutes accounted for nearly 90% of the total gut microbiota. At the genus level, the distribution proportion of several gut microbiota abundance in the three groups was statistically different (*q < *0.05) (Supplementary Table S1). Through the calculation, the Firmicutes/Bacteroidetes ratio of MDD group, Follow-up group and Controls group were 0.64, 0.46, and 0.70, respectively. The ratio in Follow-up group was significantly lower than those of the other two groups. There were significant differences among the three groups (*p* < 0.05) (Fig. 1).

#### Bacterial composition comparisons before treatment

Between the MDD and Controls groups, there were significant differences in the abundance of multiple gut microbiota at the genus level. The abundance of Parasutterella, Prevotella_9, Fusobacterium, Prevotella_2, Christensenellaceae_R-7_group, Odoribacter and [Eubacterium] _ruminantium_group significantly decreased in MDD group. Meanwhile, The abundance of Parabacteroides, Lactobacillus, Anaerostipes, and Ruminococcaceae_UCG-014 significantly increased in MDD group (*q* < 0.05) (Supplementary Table S2).

#### Bacterial composition comparisons after treatment

After escitalopram treatment, the abundance of Christensenellaceae_R-7_group, [Eubacterium] _ruminantium_group and Fusobacterium significantly increased in Follow-up group (*q* < 0.05). The abundance of Lactobacillus significantly decreased in the Follow-up group (*q* < 0.05). The main change of gut microbiota abundance in Follow-up group was Bacteroides (Supplementary Table S2).

In addition, there were also several differences in the gut microbiota between Follow-up group and Controls group. In Follow-up group, the abundance of Parabacteroides, Prevotellaceae, Ruminiclostridium_6, Flavonifractor significantly increased (*q* < 0.05), while that of Prevotella_2, Lachnospira, Collinsella, and Clostridium_sensu_stricto_1 significantly decreased (*q* < 0.05). Moreover, the abundance of Faecalibacterium and Lachnoclostridium in Follow-up group or in MDD group was significantly lower than that of the Controls group (*q* < 0.05) (Supplementary Table S2).

### Diversity analysis

#### Alpha diversity comparisons among three groups

**Figure 2:**
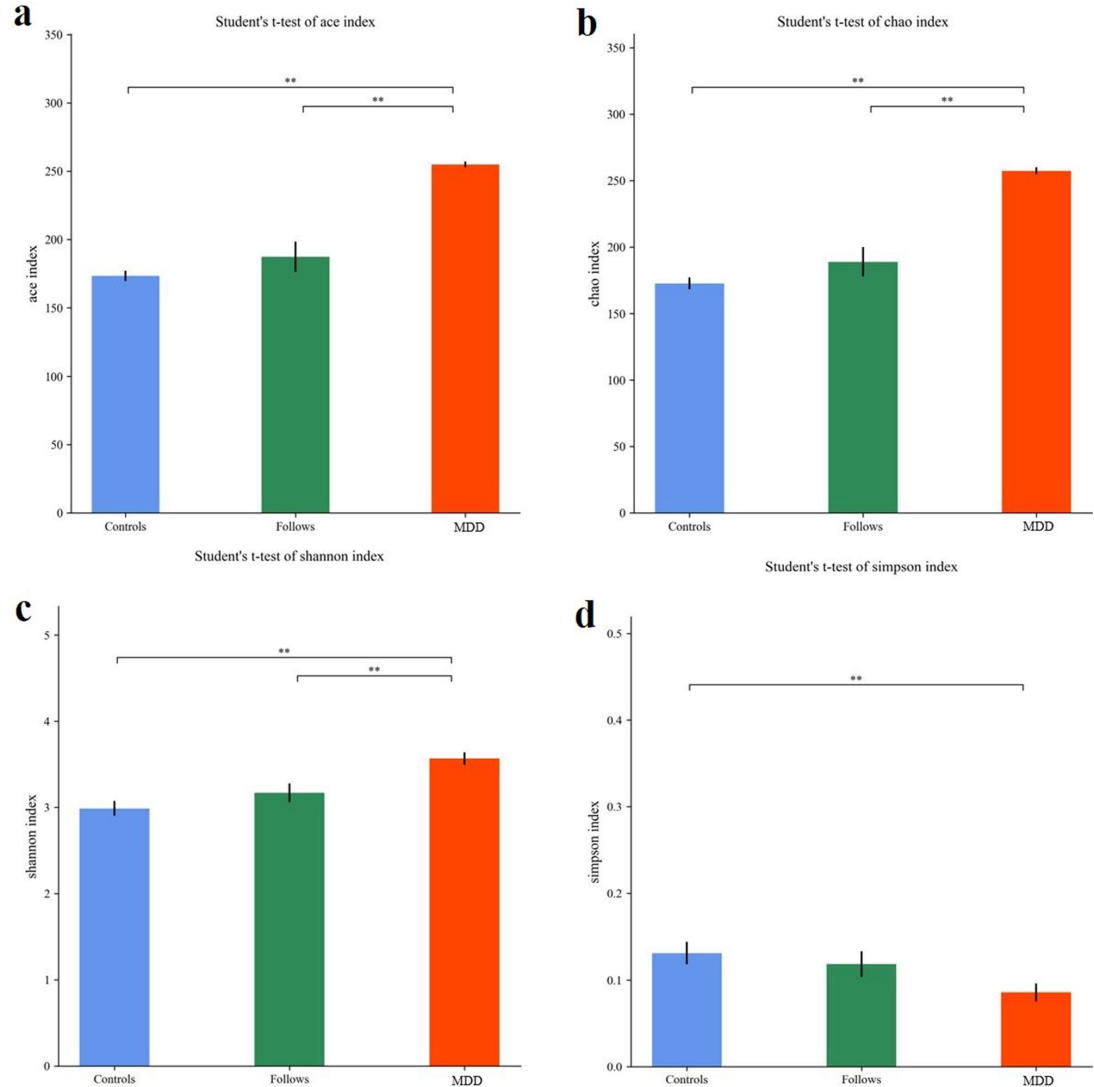
Visualization of Alpha diversity index. (**a**) Ace index; (**b**) Chaos1 index; (**c**) Shannon index; (**d)** Simpson index. **means the statistical difference between the two groups, p < 0.05.

Alpha diversity mainly reflected the richness and diversity of the species in samples. As shown in Fig. 2, indices of Chao 1, Ace, and Shannon of MDD group were significantly higher than those of Follow-up group and Controls group, and the Simpson index was significantly lower in MDD group than others. This showed that the richness and the diversity of gut microbiota in MDD group were significantly higher than those of Follow-up group and Controls group. Indices value of alpha diversity in Follow-up group was significant different from that of MDD group, and there was no significant difference between Follow-up group and Control group. The Alpha diversity of gut microbiota in patients had a tendency to return to normal. The statistics of Alpha diversity index values of each group were shown in Table 2.

**Table 2:** Richness and diversity index values of MDD, follow-up and controls.

	MDD (mean ± SD)	Follow-up (mean ± SD)	Controls (mean ± SD)
Ace	254.88 ± 2.30	187.39 ± 11.09	173.43 ± 3.80
Chaos1	257.38 ± 2.63	188.93 ± 11.07	172.69 ± 4.46
Shannon	3.57 ± 0.07	3.17 ± 0.11	2.99 ± 0.09
Simpson	0.09 ± 0.01	0.12 ± 0.01	0.13 ± 0.01

Mothur (version v.1.30) software was used to calculate the Alpha diversity index for samples. The larger of the index values of Ace and Chaos1 indices showed the greater number of species in the samples. The larger of the Shannon index value and the smaller of the Simpson index value showed more species categories of samples.

#### Beta diversity comparisons among three groups

**Figure 3:**
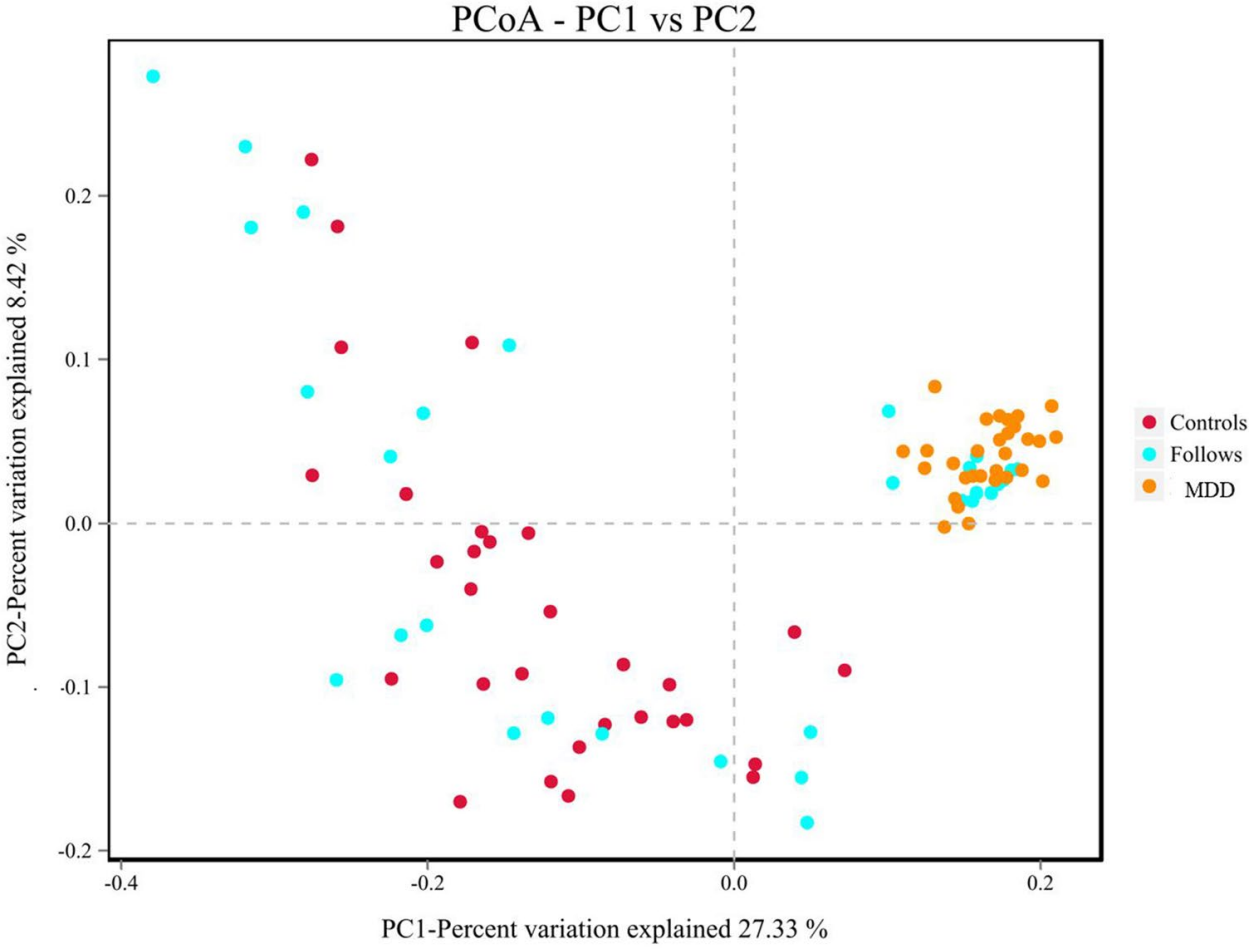
PCoA analysis shows the distribution coordinate diagram of samples: in the graph, the distance between the dots represents the similarity of the samples. Samples with high similarity tend to cluster together. The yellow dots represent the patient group, the blue dots represent the follow-up group, and the red dots represent the control group. The results showed a statistically significant difference among the three groups (R = 0.273, p = 0.001).

Beta diversity was used to compare the similarity of species diversity among different groups. The binary jaccard algorithm was used to calculate beta diversity. The gut microbiota of MDD group was significantly different with that of controls group, and the gut microbiota within MDD group was more similar (R = 0.273, *p* = 0.001) (Fig. 3).

In addition, the gut microbiota profiles of some patients treated with escitalopram were more similar to those of the control group, but the others’ profiles remained closer to those of patients. The other analysis methods employed in this study also produced similar results. The unweighted paired average method (UPGMA) was used in the R language tool to perform hierarchical clustering of each groups. It found that the gut microbiota of MDD group was significantly different with that of controls group, and the gut microbiota of follow-up group was more similar with that of controls group (Supplementary Figure S1).

#### The correlation of gut microbiota among the Follow-up group and other groups

**Figure 4:**
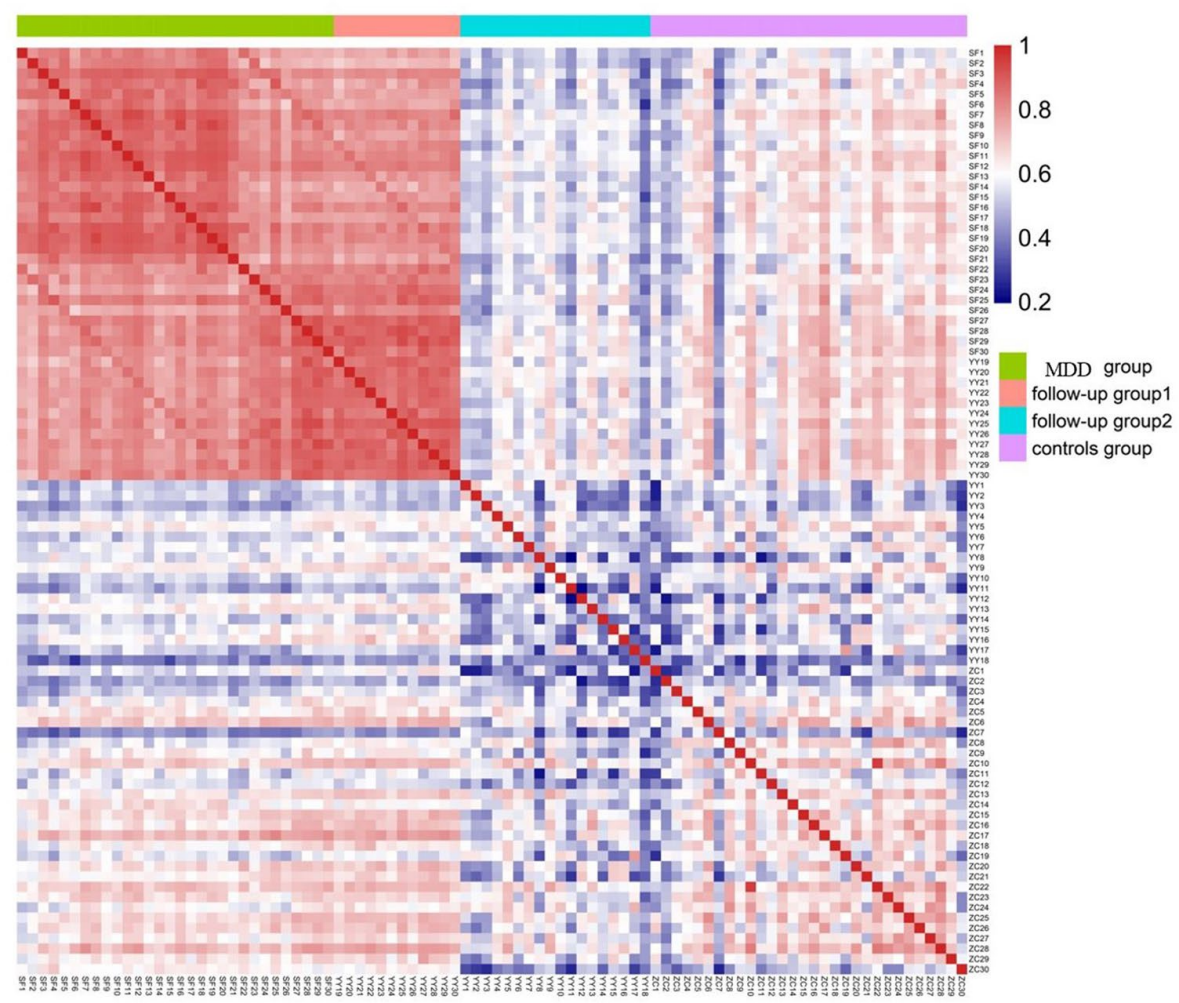
Spearman correlation coefficient heat map between samples. The closer the calculated Spearman correlation coefficient is to 1, the redder the color in the heat map, indicating the stronger correlation between the two samples. Follow-up group 1 had high correlation with Controls group. Follow-up group 2 had high correlation with MDD group.

Spearman correlation coefficient between samples was calculated to draw the heatmap. The closer of the calculated Spearman correlation coefficient was to 1, the redder of the color was in the heat map, thus indicating the stronger correlation between two samples. As shown in Fig. 4, the follow-up group could be divided into two subgroups. The gut microbiota profiles of some of the treated patients in the follow-up group remained similar to those in the MDD group, while others’ profiles were more similar with those of Controls group. These results suggested that antidepressant drugs could transform the gut microbiota of some patients into that of the control group. LEfSe was used for the quantitative analysis of biomarkers in two subgroups (LDA > 4). Several microorganisms could be selected as biomarkers in two subgroups. Gut microbiota of follow-up group 2, the subgroup associated with MDD group, was differently enriched with p_Bacteroidetes, o_Bacteroidales, c_Bacteroidia and g_Prevotella_9. While gut microbiota of follow-up group 1, the subgroup associated with Controls group, was differently enriched with p_Firmicutes, p_Actinobacteria, f_Lachnospiraceae, f_Bifidobacteriaceae, o_Bifidobacteriales, c_Actinobacteria and g_Bifidobacterium (Supplementary Figure S2).

### Functional properties predicted by PICRUSt

**Figure 5:**
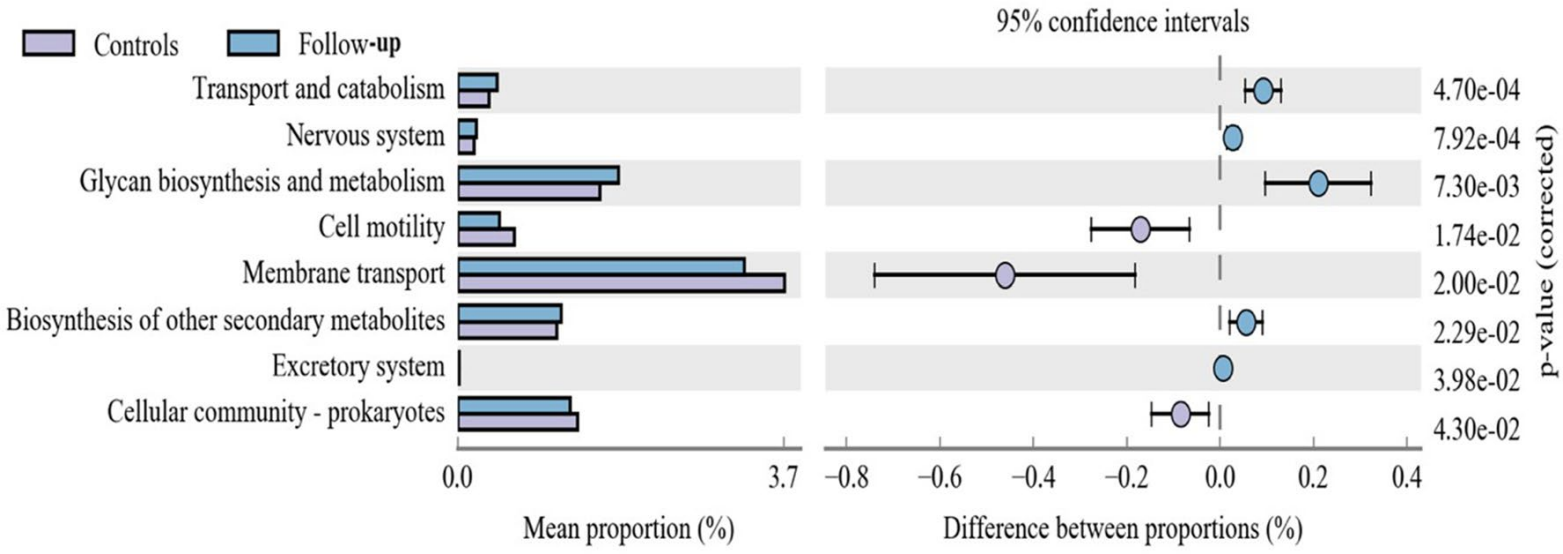
Metabolic pathway analysis between Controls group and Follow-up group. The left side of the figure shows the abundance ratio between the two groups. The middle section shows the proportional variation in functional abundance within the 95% confidence interval. The p value is on the right.

The study considered that the profiles of gut microbiota in follow-up group could not completely return to the normal state. The PICRUSt software was used to compare the species composition information obtained from 16S sequencing data to infer the functional gene composition between patients and controls. Through the annotation of the KEGG metabolic pathway, it found that there were differences in the metabolic pathways of Transport and catabolism, Nervous system, Glycan biosynthesis and metabolism, Cell motility and Membrane transport between Follow-up group and Controls group (*p* < 0.05)(Fig. 5). In MDD group and Controls group, the major different pathways included Glycan biosynthesis and metabolism, Transport and catabolism, Excretory system, Metabolism of other amino acides, and Nervous system, which were similar with the result of those of Follow-up group and Controls group (Supplementary Figure S3). Meanwhile, only the pathway of Biosynthesis of other secondary metabolites was significantly differences between MDD group and Follow-up group (Supplementary Figure S4). It suggested that the gut microbiota of Follow-up group might still involve in the occurrence of depression.

## Discussion

This study demonstrated that the gut microbiota from patients of drug-naive first-episode MDD was significantly different with that of controls, and the composition and structure of the gut microbiota in patients were more similar. This suggested that the occurrence and development of MDD might be associated with specific gut microbiotas. However, the identification results of gut microbiota structure were inconsistent^7, 20, 21^. In the study of Lin et al., they found the MDD patients had more phylum Firmicutes and less Bacteroidetes^7^. On the contrary, Huang et al. reported that Firmicutes were significantly lower in patients with depression^21^. The inconsistency of the results from different studies may be related to factors including small sample size, different inclusion criteria, course, severity of disease, etc.^22^. However, some studies have shown that transplanting fecal samples from depressed patients could induce depressive symptoms in mice^23, 24^. This suggested that a disturbed gut microbiota could be one of the causes of depression.

There is still a lack of effective biomarkers as clinical guidance for the diagnosis and treatment of depression^25^. Several studies have shown that gut microbiota, used as a biomarker, has a good distinguishing effect on depression, AUC value 0.702-0.986^26–28^. Gut microbiota profiles can be used to effectively distinguish not only patients from normal population, but also major depressive disorder and bipolar depression, and even evaluate the therapeutic effect as well^27, 28^. In this study, the major changed gut microbiota were Bacteroidetes. Monitoring the variation of Bacteroidetes may indicate the prognosis of the disease and the efficacy of the drug, which could be used as a potential biomarker.

In 2011, Manimozhiyan Arumugam et al. proposed the concept of enterotypes^29^. In the sequencing results, they reported that the human gut microbiome could be divided into three robust clusters, including Prevotella-enterotype (enterotype P), Bacteroides-enterotype (enterotype B) and Ruminococcus-enterotype (enterotype R). Different enterotypes have characteristic advantages of respective functional states^30^. For example, enterotype P can generate more short-chain fatty acids and thus has stronger fermentation ability. Enterobacter type B has a variety of specific enzymes, which can promote the hydrolysis of sugars and proteins in food and improve the absorption by the body^31^. Our study found that the composition of gut microbiota in Follow-up group resembled participants in the control group, but the metabolic function of gut microbiota in Follow-up group was still similar to that of MDD group. We considered that the partial composition of gut microbiota had changed after treatment, but the enterotype was not, so that it still retained abnormal metabolic characteristics. However, we were unable to confirm this hypothesis due to short follow-up period in this study. The Belgian Flemish Gut Flora Project have found that enterotype distribution varied with depression status^32^. Therefore, the future study could explore the relationship between enterotypes and metabolic function in different period of depression, which may help to understand the impact of gut microbiota on the development of MDD.

In addition, the gut microbiota tended to “normal” gut microbiota structure under SSRIs treatment, thus indicating a positive effect of SSRIs on the change of gut microbiota. However, there is currently no consensus on the effect of antidepressants on the gut microbiota. A recent study reported Lachnospiraceae species were more abundant in SSRIs treated mice compared to controls^33^. However, the opposite conclusion was reported in Valles-Colomer et al*.*’s study^32^. Since gut microbiota is easily affected by a variety of factors, future research should pay attention to course of disease, severity, population characteristics and other aspects to further clarify the change of gut microbiota.

The outcomes of current study were limited to the relatively small sample size and short follow-up time. This study was unable to further observe the effects of different antidepressant doses on the composition of gut microbiota. The effect of diet on gut microbiota was also not fully considered. Different diets and microbial combinations have different effects on the physiological function and substance metabolism of the intestinal tract^34^. In the future, we could further expand the sample size for research. Based on the understanding of the changes of gut microbiota, the correlation between gut microbiota and clinical phenotypes could be further elucidated. Moreover, metabolomics and proteomics omics technologies are strongly suggested to be employed to explore the relationship between gut microbiota and depression.

## Conclusions

In this study, it found that the gut microbiota of patients with first-episode depression was significantly different with that of Controls group. After escitalopram treatment, gut microbiota diversity of depressive patients tended to return to the normal state. However, there were still several structures and metabolic pathways difference in the gut microbiota between follow-up patients and controls, which might be related to the relapse of depression.

## Methods

### Participants

The 60 local subjects, including 30 depressed patients and 30 normal subjects, were recruited in this study. The inclusion criteria for subjects described as follows: (1) age was between 18–65 years old; (2) body mass index (BMI) was between 18 and 28 kg/m^2^; (3) no history of treatment with antipsychotic medication; (4) duration of symptoms was between 1 and 24 months; (5) no history of treatment with antidepressant medication; (6) currently in the acute episode with the Hamilton Depression Rating Scale for Depression (HAMD) score ≥ 24; (7) other mental disorders such as axis I, personality disorder and mental retardation were excluded; (8) psychotropic drugs were never used; (9) diagnosis of depression in MDD group was made by two psychiatrists according to the Mini-International Neuropsychiatric Interview (MINI); (10) in Controls group, a diagnosis of mental disorder was excluded by two psychiatrists according to the MINI, and HAMD-17 score was < 7; (11) MDD group was defined as Follow-up group after receiving the drug treatment.

In addition, a series of exclusion criteria, based on the previous work, were employed in this study to exclude the factors affecting gut microbiota^35^. Those include: (1) no somatic diseases known to affect the gut microbiota such as inflammatory bowel disease, immune system diseases, diabetes, etc.; (2) without antibiotics, probiotics or microbiological products used in recent 3 months; (3) no history of medical examination or surgery through the gastrointestinal tract in recent 6 months; (4) without obvious changes in dietary habits or the presence of obvious diarrhea, constipation and other symptoms in recent 1 month.

According to the questionnaire, subjects’ life events that may affect the mood, such as examinations, unemployment and bereavement during the last six months and the whole research period, were surveyed and recorded. All of subjects in this study were required to sign an informed consent. According to the Helsinki Declaration, the protocol for sample collection and analysis was approved by the Ethics Committee of Peking University Sixth Hospital and Beijing Hospital of Chinese Traditional and Western Medicine.

### Sample collection

Fecal samples were obtained from subjects enrolled. Subjects were instructed by staff to discharge feces into a clean container. After defecation, the staff collected 2 g fecal sample and quickly placed it into a container containing liquid nitrogen. The samples were then frozen at − 80 °C until analysis.

### Treatment

All patients with depression received individualized treatment with escitalopram. The starting dose of escitalopram was 5 mg/day from day 1-day 7 and increased to 10 mg/day from day 8. According to the individual response, the dose of escitalopram could be adjusted, and the maximum dose was 20 mg/day. After 4–6 weeks of treatment, the patients were evaluated by HAMD scale. When the scale reduction rate of HAMD was ≥ 50% compared with baseline, their fecal sample was collected for the second time and recorded as ‘Follow-up group’ to be used in the comparison of gut microbiota.

### 16S rRNA Amplification of V3-V4 region and Illumina Sequencing

Using a PowerSoil DNA kit (MoBio, USA), DNA extraction was performed from 200 mg fecal samples according to manufacturer's instructions. KAPA HiFi HotStart ReadyMix (KAPA, USA) was used to amplify the 16S rRNA (V3–V4) gene marker. Each DNA sample of the bacterial 16S rRNA gene was amplified with primers 341F (GGACTACHVGGGTWTCTAAT) and 805R (ACTCCTACGGGAGGCAGCAG). The primers included a unique 8-nucleotide barcode and an Illumina adapter. Polymerase chain reaction (PCR) conditions were set as follows: initial denaturation at 95 ℃ for 5 min, 98 ℃ denaturation for 20 cycles for 20 s, 58 ℃ annealing for 30 s, 72 ℃ extension for 30 s, and 72 ℃ final extension for 5 min. The amplicons obtained by PCR were analyzed on 1.5% agarose gel electrophoresis, and a band of a desired size was purified using a QIAquick gel extraction kit (QIAGEN, Germany). The product was submitted to the second-generation sequencing laboratory of Beijing institute of bioinformatics for sequencing on Illumina HiSeq 2500 platform.

### Bioinformatics analysis

The QIIME (Version 1.9.1) software was used to filter and sequence the original sequence to obtain optimized sequences (Tags)^36^. Fragments containing ambiguous characters in the sequence or more than two nucleotide mismatched primers were removed. Usearch (version 10.0) software was used to cluster Tags at a similarity level of 97% to obtain OTUs^37^. OTUs were annotated based on the Silva (bacterial) and UNITE (fungi) taxonomy databases. QIIME (Version 1.9.1) software was used to generate species richness tables at different taxonomic levels, and R language tools were used to draw community structure maps at each taxonomic level of the sample. The community structure map of each sample was obtained at the level of taxonomy, class, order, family, genus, species.

In order to identify the difference in microbial community richness between the MDD group and Controls group, the Metastats (URL: http://metastats.cbcb.umd.edu) software was used to perform a T test on the species richness data between two groups to obtain the *p* value^38^. The *q* value was obtained by correcting the *p* value. Species were selected based on p values or *q* values that caused differences in the composition of the two groups of samples. The analysis was performed at the level of phylum, class, order, family, genus, species taxonomy to analyze the significance between groups.

Mothur (version v.1.30, URL: http://www.mothur.org/) software was used to evaluate the Alpha Diversity Index of the samples^39^. The species diversity within a single sample was studied by Alpha Diversity Analysis, and the Ace, Chao1, Shannon, and Simpson indices of each sample at the 97% similarity level were counted; Beta diversity analysis was performed using QIIME (Version 1.9.1) software. Beta diversity analysis mainly used the binary jaccard algorithm to calculate the distance among samples to obtain the β value between samples. Based on the distance matrix obtained from the Beta diversity analysis, PCoA analysis was performed using R language tools to further demonstrate the differences in species diversity among samples^40^. Hierarchical clustering was performed on samples using unweighted paired average method (UPGMA) to determine the similarity of species composition among samples. According to the species abundance table obtained by clustering, spearman correlation coefficient among samples was calculated by Psych package in R language, and then the heatmap was drawn by P heatmap package in R language. The closer of the calculated Spearman correlation coefficient was to 1, the darker the red shading was in the heat map, thus indicating the stronger correlation of two samples. Then, LEfSe tools (URL: http://huttenhower.sph.harvard.edu/lefse/) were used the Wilcox test function of the R language STATS package to estimate the impact of the abundance of each component (species) on the effect of the difference between components, so that the comparison of two subgroups can be realized to find the species marker (Biomaker) with significant difference in the abundance^41^.

PICRUSt (Version 1.1.4) software was used to compare the species composition information obtained from 16S sequencing data to deduce the functional gene composition in the samples, thereby determining the functional differences between different groups^42^. Using the KEGG orthology database (KOs) in the Kyoto Encyclopedia of Genes and Genomics (KEGG) database^43^, the changes in metabolic pathways of functional genes of microbial communities between different groups were evaluated through differential analysis of KEGG metabolic pathways^44, 45^.

### Statistics analysis

Statistical analysis was performed using SPSS19.0 software. Participants' gender, tobacco and alcohol consumption were expressed in terms of proportional or percentages. Independent t tests, Welch t tests, and White non-parametric t tests were used for continuous variables. Pearson chi-square test or Fisher's exact test were used for classification variables. All significance tests were two-sided tests, and p < 0.05 or adjusted p < 0.05 was considered statistically significant.

## Supplementary Information

Supplementary Information 1.
