# Supplementary material for: The change of gut microbiota in MDD patients under SSRIs treatment
Source: Sci Rep. 2021 Jul 21;11:14918. doi: 10.1038/s41598-021-94481-1 (PMC8295378; doi:10.1038/s41598-021-94481-1)
Supplement: Supplementary file 1 — Supplementary Information 1. [file 41598_2021_94481_MOESM1_ESM.pdf]

The change of gut microbiota in MDD patients under SSRIs treatment

Yang Shen[1*], Xiao Yang[2*], Gaofei Li[3*], Jiayu Gao[4#]， Ying Liang1[#]

1. National Clinical Research Center for Mental Disorders, Peking University Sixth Hospital, Institute of Mental Health, Key Laboratory of Mental Health, Ministry of Health, Peking University, Beijing, China;

2. School of Clinical Medicine, Henan university of science and technology;

3. Department of Psychiatry, Beijing Hospital of Chinese Traditional and Western Medicine, Beijing, China;

4. School of Chemical Engineering and Pharmaceutics, Henan university of science and technology.

# Correspondence to: J.Y. Gao, School of Chemical Engineering and Pharmaceutics, Henan university of science and technology，No. 263, Kaiyuan Boulevard, Luolong District, 471023, Luoyang City, Henan Province, China. E-mail addresses: j.gao@haust.edu.cn (Jiayu Gao).

Correspondence to: Y. Liang, National Clinical Research Center for Mental Disorders, Peking University Sixth Hospital, Institute of Mental Health, Ministry of Health, Peking University, Haidian District, Huayuanbeilu 51, 100191, Beijing, China. E-mail addresses: liangying1980@bjmu.edu.cn (Ying Liang). Telephone numbers：+086-13581827654

* These authors contributed equally to this work.

Supplementary Figure Legends

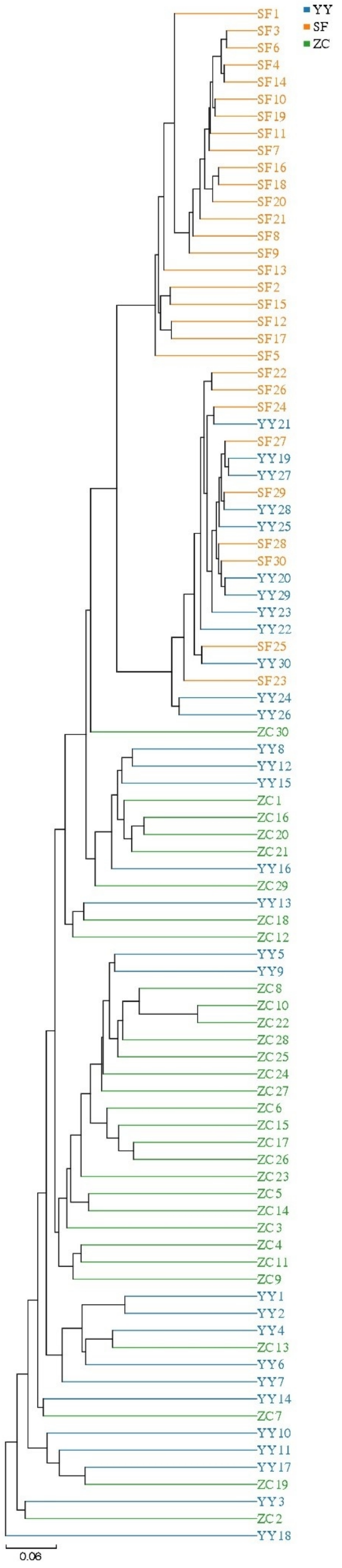

Supplementary Figure S1.

Supplementary Figure S1. Sample clustering tree. It has shown that two samples of species were more similar to the branches was shorter and closer. In this Figure, the first episode depressive patients were named SF; patients for treatment with ecitalopram were named YY； health controls were named ZC. The gut microbiota of depressed patients was significantly different from that of the normal population and was highly similar. After treatment, the microbiota of depressed patients was more similar to that of normal people.

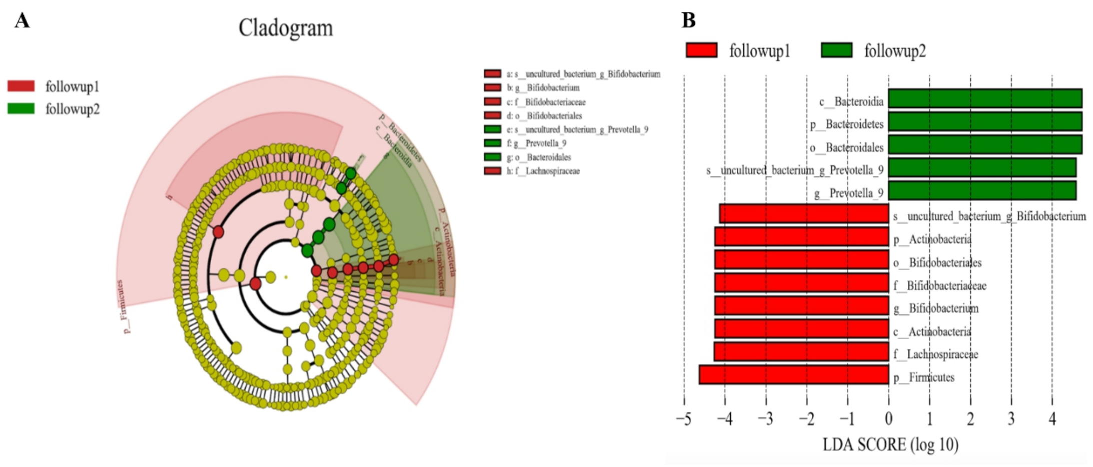

Supplementary Figure S2

Supplementary Figure S2. Differently abundant taxa identified using LEfSe analysis by QIIME (Quantitative Insights Into Microbial Ecology, Version 1.9.1) software. A. LEfSe cladogram showed the most differentially abundant taxa between the two cohorts. Taxa enriched for follow-up group1 in red; follow-up group 2 enriched taxa in green. The brightness of each dot is proportional to its effect size. B. Visualization of only taxa meeting an LDA threshold >4.

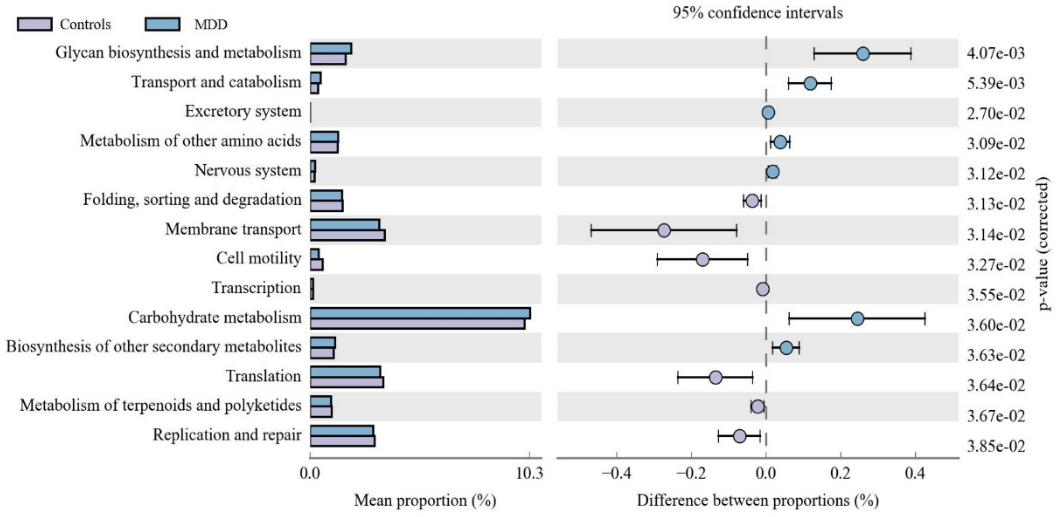

Supplementary Figure S3

Supplementary Figure S3. Metabolic pathway analysis between Patients group and Controls group. The left side of the figure shows the abundance ratio between the two groups. The middle section shows the proportional variation in functional abundance within the 95% confidence interval. The p-value is on the right.

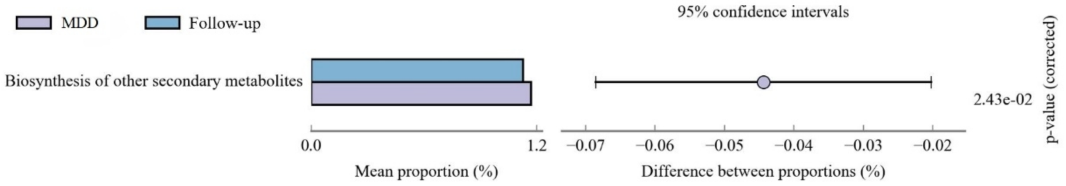

Supplementary Figure S4

Supplementary Figure S4. Metabolic pathway analysis between Patients group and Follow-up group. The left side of the figure shows the abundance ratio between the two groups. The middle section shows the proportional variation in functional abundance within the 95% confidence interval. The p-value is on the right.

Supplementary table

Supplementary table S1. Differences in gut microbiota among three groups at phylum level

	Mean(Patient)	Mean(Control)	Mean(Follow)	*p*-value	*q*-value
Firmicutes	0.33±0.12	0.36±0.13	0.28±0.12	0.02*	0.04*
Bacteroidetes	0.55±0.14	0.54±0.14	0.64±0.14	0.01*	0.03*
Actinobacteria	0.02±0.03	0.02±0.02	0.02±0.07	0.95	0.95
Proteobacteria	0.07±0.05	0.06±0.05	0.05±0.03	0.48	0.66

*The difference was significant. $p<0.05$ or $q<0.05$.

Supplementary table S2. Differences in gut microbiota among three groups at genus level

	Mean(Patient)	Mean(Control)	Mean(Follow)	*p*-value	*q*-value
Family_XIII_UCG-001	0.00±0.00	0.00±0.00	0.00±0.00	< 0.01*	0.01*
Ruminococcaceae_UCG-003	0.00±0.00	0.00±0.00	0.00±0.00	< 0.01*	0.02*
Ruminococcaceae_UCG-014	0.02±0.02	0.00±0.01	0.00±0.01	< 0.01*	0.03*
Bacillus	0.00±0.00	0	0.00±0.00	< 0.01*	0.01*
Lachnospira	0.01±0.01	0.02±0.02	0.01±0.01	< 0.01*	< 0.01*
Dorea	0.00±0.00	0.01±0.00	0.00±0.00	< 0.01*	0.01*
Parasutterella	0.03±0.04	0.01±0.01	0.01±0.01	< 0.01*	0.01*
Collinsella	0.00±0.00	0.00±0.00	0.00±0.00	0.01*	0.02*
[Eubacterium]_eligens_group	0.01±0.01	0.00±0.00	0.00±0.01	0.01*	0.02*
Odoribacter	0.00±0.00	0.00±0.00	0.00±0.01	0.01*	0.01*
Erysipelatoclostridium	0.00±0.00	0.00±0.00	0.00±0.00	0.01*	0.01*
Ruminococcaceae_NK4A214_group	0.00±0.00	0.00±0.00	0.00±0.00	0.01*	0.02*
Bacteroides	0.40±0.11	0.37±0.19	0.49±0.17	0.02*	0.01*
Lachnospiraceae_NK4A136_group	0.01±0.01	0.00±0.01	0.01±0.01	0.02*	0.01*
Intestinimonas	0.00±0.00	0.00±0.00	0.00±0.00	0.02*	0.02*
Unassigned	0.00±0.00	0	0.00±0.00	0.02*	0.02*
Oscillibacter	0.00±0.00	0.00±0.00	0.00±0.00	0.02*	0.02*
uncultured_bacterium_f_Lachnospiraceae	0.01±0.00	0.01±0.01	0.01±0.00	0.02*	0.02*

Granulicatella	0.00±0.00	0.00±0.00	0.00±0.00	0.04*	0.24
Hungatella	0.00±0.00	0.00±0.00	0.00±0.00	0.04*	0.25
UBA1819	0.00±0.00	0.00±0.00	0.00±0.00	< 0.05*	0.28
[Eubacterium]_hallii_group	0.00±0.00	0.01±0.01	0.00±0.00	< 0.05*	0.28
Ruminococcaceae_UCG-004	0.00±0.00	0.00±0.00	0.00±0.00	< 0.05*	0.27

*The difference was significant. p<0.05 or q<0.05.
